# Adherence to the evidence-based recommendations in managing bone health, pain, and mobility of patients with multiple myeloma: a mixed method in the Palestinian healthcare system

**DOI:** 10.1186/s12885-024-12024-z

**Published:** 2024-03-05

**Authors:** Ramzi Shawahna, Riad Amer, Husam Salameh, Abdul-Rahman Shawahna, Mohmmad Aljondy, Mohmmad Zain-Aldain

**Affiliations:** 1 Department of Physiology, Pharmacology and Toxicology, Faculty of Medicine and Health Sciences, An-Najah National University, New Campus, Building: 19, Office: 1340, Nablus, P.O. Box 7, Palestine; 2 Clinical Research Center, An-Najah National University Hospital, 44839 Nablus, Palestine; 3 Department of Medicine, Faculty of Medicine and Health Sciences, An-Najah National University, Nablus, Palestine; 4 Hematology and Oncology, An-Najah National University Hospital, 44839 Nablus, Palestine

**Keywords:** Bone health, Pain, Mobility, Multiple myeloma, Quality of care

## Abstract

**Background:**

Consensus/evidence-based recommendations for assessing, managing, and monitoring bone health, pain, and mobility in patients with multiple myeloma were developed. This study was conducted to assess the adherence of the hematologists-oncologists to the consensus/evidence-based recommendations for assessing, managing, and monitoring bone health, pain, and mobility in patients with multiple myeloma who received care in the Palestinian healthcare system.

**Methods:**

A mixed method was used in this study. The consensus/evidence-based recommendations were identified through a systematic search in Scopus, PubMed, SpringerLink, ScienceDirect, and Google Scholar. A panel of 5 researchers (3 hematologists-oncologists, 3 medical students, and 1 pharmacologist) sorted the consensus/evidence-based recommendations and developed the survey tool during 3 iterative meetings. The extent to which the hematologists-oncologists in the 5 centers caring for patients with multiple myeloma adhered to the consensus/evidence-based recommendations was assessed using a questionnaire.

**Results:**

Responses were collected from 10 hematologists-oncologists in all 5 healthcare centers where patients with multiple myeloma receive healthcare in the West Bank of Palestine. The median number of years in the practice of the hematologists-oncologists was 7.5 [2.75, 14.0] years and the median number of patients with multiple myeloma care per month was 12.5 [7.5, 21.25]. The vast majority (90%) of the hematologists-oncologists reported inadequate adherence to screening for medication problems related to bone health, pain, cardiopulmonary fitness, healthy behaviors, nutritional deficits, and mental health. Of the hematologists-oncologists, 70% reported inadequate adherence to ordering and evaluating calcium, vitamin D, alkaline phosphatase, electrolytes, and phosphorus levels to monitor bone health and 60% reported inadequate adherence to prescribing calcium and vitamin D supplements whenever there was a need.

**Conclusion:**

The findings of this study suggested inadequate adherence to the consensus/evidence-based recommendations and highlighted areas for improvement to ensure that patients receive optimal care. The findings suggested a need for further education and training on the latest guidelines and recommendations. Decision-makers and policymakers might need to design measures and implement policies to improve adherence to the consensus/evidence-based recommendations. Addressing these gaps in adherence to the consensus/evidence-based recommendations may improve the care and outcomes of patients with multiple myeloma.

## Introduction

Multiple myeloma is an incurable hematological malignancy in which clonal plasma cells proliferate in the bone marrow. Multiple myeloma is the second most common hematological malignancy. Worldwide, multiple myeloma accounts for about 13% of all hematological cancers [1]. The incidence of multiple myeloma was estimated at 6.1 per 100,000 people per year [2, 3].

It is well-established that multiple myeloma can cause significant osteolytic and osteoporotic bone disease [4, 5]. In addition, multiple myeloma affects older adults, among whom, osteoporosis is also common [6]. Therefore, bone disease is a major source of morbidity and mortality among patients with multiple myeloma [5]. It has been suggested that osteolytic lesions develop as a result of increased osteoclastic resorption [4, 5, 7]. Additionally, increased bone resorption results from the interaction between bone marrow stromal cells and myeloma tumor cells within the bone marrow microenvironment [7]. Therefore, the overproduction of osteoclasts and reduced stimulation of osteoblasts leads to imbalanced bone turnover and increased risk of pathologic fractures [8–10]. Stimulation of osteoclast-activating growth factors, cytokine release, and lack of osteoblastic response can lead to the development of destructive bone lesions and diffuse osteopenia [4, 5, 7, 11]. The consequences of these osteolytic bone lesions can be severe for patients, causing fractures, severe bone pain, spinal cord compression, hypercalcemia, and renal insufficiency, all of which can have a devastating effect on the quality of life and worsen survival prospects for patients [10, 12].

Previous studies have reported that the vast majority (about 85%) of patients with multiple myeloma develop bone disease [7, 13]. It is well-established that lytic bone lesions are associated with fractures, poor mobility, pain, poor blood circulation, and incidence of blood clots. Therefore, hematologists-oncologists and other healthcare professionals caring for patients with multiple myeloma should address bone health while diagnosing, caring for, and following up with this particular group of patients.

Different professional groups and associations including the International Myeloma Working Group (IMWG) have developed consensus/evidence-based recommendations to guide assessing, managing, and monitoring bone health, pain, and mobility among patients with multiple myeloma [5, 10, 14–17]. These recommendations can be used at varying points in the trajectory of the disease.

In Palestine, little is known about the adherence of the hematologists-oncologists to the consensus/evidence-based recommendations for assessing, managing, and monitoring bone health, pain, and mobility in patients with multiple myeloma. Therefore, this study was conducted to assess the adherence of the hematologists-oncologists to the consensus/evidence-based recommendations for assessing, managing, and monitoring bone health, pain, and mobility in patients with multiple myeloma who received care in the Palestinian healthcare system. The findings of this study might be informative to decision-makers and policymakers who might be interested in improving care for patients with multiple myeloma, maintaining bone health, reducing pain and other disabling symptoms, promoting mobility, and improving the health-related quality of life of the affected patients. The findings of this study might also apply to other healthcare systems in poor, underdeveloped, and resource-limited settings.

## Methods

### Study design

In this study, a mixed method that combined qualitative interviews and a survey to assess the adherence of the hematologists-oncologists to the consensus/evidence-based recommendations for assessing, managing, and monitoring bone health, pain, and mobility in patients with multiple myeloma who receive care in the Palestinian healthcare system.

#### Development of the survey tool

The survey tool was developed following a review of the consensus/evidence-based recommendations used for assessing, managing, and monitoring bone health, pain, and mobility in patients with multiple myeloma [1–3, 6, 10, 14, 16, 18–46]. A panel of 5 researchers (3 hematologists-oncologists, 3 medical students, and 1 pharmacologist) developed the survey tool during 3 iterative meetings [47]. In the first meeting, the retrieved consensus/evidence-based recommendations were sorted, summarized, and distributed to the panelists. In the second meeting, a draft survey tool that included the consensus/evidence-based recommendations to screen for was developed. In the third meeting, consensus was achieved on the final form of the survey tool. The panelists were asked to rate each included item based on its clarity, relevance, and suitability using a scale of 1 to 5 (where 1 indicated not clear/relevant/suitable at all, and 5 indicated very clear/relevant/suitable). The items that received a rating of clear/relevant/suitable or very clear/relevant/suitable were included in the final survey tool. Any disagreements were resolved through discussion and consensus.

### Study settings

This study was conducted in the main centers where patients with multiple myeloma receive healthcare services in the Palestinian healthcare system in the West Bank. At the time of the study, patients with multiple myeloma received healthcare services in 5 main centers that were located in the north, center, and south of the West Bank: Nablus (*n = 2*), Jerusalem (*n = 1*), Ramallah (*n = 1*), and Beit Jala (*n = 1*).

Patients suspected of having multiple myeloma are referred to one of the 5 centers. At the time of the study, the 5 centers used the revised IMWG criteria for the diagnosis of multiple myeloma [1, 16, 29–36]. Patients are assessed for the classically used CRAB features: hypercalcemia, renal insufficiency, anemia, and destructive bone lesions. In addition, the revised IMWG criteria also allowed 3 myeloma-defining events. These myeloma-defining events were: (1) clonal bone marrow plasma cells ≥ 60%, (2) serum involved/uninvolved free light chain ratio ≥ 100, provided the absolute level of the involved light chain ≥ 100 mg/L (kappa or lambda > the normal reference range), and 3) > 1 focal lesion of ≥ 5 mm on magnetic resonance image. Diagnosis of multiple myeloma is made based on the presence of at least 1 of these markers, regardless of the absence or presence of CRAB features or other symptoms. Active multiple myeloma is defined by: (1) clonal bone marrow plasma cells of more than 10%, or biopsy-proven bony, or extramedullary plasmacytoma, and (2) presence of 1 or more of the CRAB features or the myeloma-defining events.

For the diagnosis of multiple myeloma, the following procedures, laboratory testing, and imaging should be performed or ordered [1, 16, 29–36]: (1) history and physical examination, (2) bone marrow examination to look for clonal plasma cells, biopsy-proven bone, or soft tissue (extramedullary) plasmacytoma, (3) complete blood count with differential and peripheral blood smear review, (4) blood chemistry panel, kidney function tests, liver function tests, and blood calcium, (5) proteins and monoclonal proteins, and (6) bone imaging surveys.

The patients are treated after considering the disease stage, patient’s health and age, genetics and molecular markers, prior treatments, goals of treatment, tolerability of treatment, and adverse effects [10, 16, 34]. The patients might receive chemotherapy, radiotherapy, immunomodulatory medications, proteasome inhibitors, corticosteroids, and monoclonal antibodies. Eligible patients are considered for autologous stem cell transplantation. In addition, patients receive supportive care to manage pain, minimize adverse effects, maintain bone health, and improve mobility, physical activity, mental health, and well-being of the patients. Patients are periodically assessed, evaluated, and monitored before, during, and after treatments. The frequency of these assessments, evaluations, and monitoring can vary between the patients by the disease stage and response to the treatment. Depending on the treatment, patients are scheduled for a follow-up visit on a weekly, every other week, or monthly.

### Data collection and analysis

The survey tool was completed by 2 hematologists-oncologists from each center during an interview. One of the 2 hematologists-oncologists was the head of the hematology/oncology department of each center and the second was a main hematologist-oncologist. Three final-year medical students interviewed the hematologists-oncologists in quiet places/rooms in the centers. The medical students were trained to conduct interviews specifically for this study by the main investigator (RS) who had extensive experience in conducting qualitative interviews. The hematologists-oncologists were asked to provide their gender, age, and the center at which they practiced. In addition, the hematologists-oncologists were asked to provide their practice-related variables like the number of years in practice, the number of patients cared for per month, and the number of patients with multiple myeloma cared for per month. The survey tool contained 6 items related to the diagnosis of patients with multiple myeloma. These items were based on the revised criteria of the IMWG for the diagnosis of multiple myeloma [1, 16, 29–36]. In addition, the tool also contained 22 items related to monitoring vital signs, hematologic issues, kidney, and liver functions (*n = 4*), monitoring and maintaining bone health (*n = 5*), monitoring and managing pain (*n = 4*), and monitoring mobility, physical activity, mental health, and well-being (*n = 9*) of patients with multiple myeloma. On each item, the hematologists-oncologists had to indicate whether the hematologists-oncologists at the center adequately or inadequately adhered to the recommended guidelines. Responses of the hematologists-oncologists were analyzed descriptively using frequencies and percentages.

### Ethical approval

The study was conducted following the principles of scientific and medical research outlined in the Declaration of Helsinki. The study protocol and ethics of this study were approved by the Institutional Review Board (IRB) of An-Najah National University. Before participating in the study, the hematologists-oncologists provided written informed consent.

## Results

In this study, responses were collected from all 5 healthcare centers where patients with multiple myeloma receive healthcare in the West Bank of Palestine. The survey tool was filled by 10 hematologists-oncologists (2 hematologists-oncologists from each center: the head of the department, and a main hematologist-oncologist). The median number of years in the practice of the hematologists-oncologists was 7.5 [2.75, 14.0] years, the median number of patients cared for per month was 225 [75.0, 300.0], and the median number of patients with multiple myeloma care for per month was 12.5 [7.5, 21.25]. The characteristics of the hematologists-oncologists are shown in Table 1.

**Table 1:** Demographic and practice characteristics of the hematologists-oncologists

Variable	n	%
**Gender**		
Male	6	60
Female	4	40
**Age (years)**		
< 40	5	50
≥ 40	5	50
**Region of practice**		
North	5	50
Center	3	30
South	2	20
**Number of years in practice, median [Q1, Q3]**	7.5 [2.75, 14.0]	
**Number of patients cared for per month, median [Q1, Q3]**	225 [75.0, 300.0]	
**Number of patients with multiple myeloma cared for per month, median [Q1, Q3]**	12.5 [7.5. 21.3]	

Q1: first quartile, Q3: third quartile

### Adherence to the recommended guidelines during the diagnosis of multiple myeloma

In this study, all the hematologists-oncologists (100%) reported adequate adherence to the guidelines by taking a medical history and conducting physical examinations during the diagnosis of patients with multiple myeloma. Similarly, the hematologists-oncologists reported adequate adherence to ordering and evaluating bone marrow examinations to look for clonal plasma cells, biopsy-proven bone, or soft tissue (extramedullary) plasmacytoma, as indicated in the recommended guidelines. Moreover, kidney function tests and hemoglobin levels were always ordered and evaluated, as indicated in the recommended guidelines. On the other hand, 50% of the hematologists-oncologists reported inadequate adherence to ordering and evaluating bone imaging to look for osteolytic lesions and 20% of the hematologists-oncologists reported inadequate adherence to ordering and evaluating blood calcium levels. Adherence of the hematologists-oncologists to the recommended guidelines for diagnosing patients with multiple myeloma is shown in Table 2.

**Table 2:** Adherence of the hematologists-oncologists to the recommended guidelines for diagnosing patients with multiple myeloma

		Adherence to the recommendations/guidelines			
		Inadequate adherence		Adequate adherence	
#	Item^*^	n	%	n	%
1	Take the patient’s medical history and conduct a physical examination	0	0	10	100
2	Order and evaluate a bone marrow examination to look for clonal plasma cells, biopsy-proven bone, or soft tissue (extramedullary) plasmacytoma	0	0	10	100
3	Order and evaluate blood calcium levels	2	20	8	80
4	Order and evaluate kidney function tests	0	0	10	100
5	Order and evaluate hemoglobin levels	0	0	10	100
6	Order and evaluate bone imaging to look for osteolytic lesions	5	50	5	50

^*^Based on [29–36, 48]

### Adherence to the recommended guidelines for monitoring patients with multiple myeloma

#### Monitoring vital signs, hematologic issues, kidney, and liver functions

In this study, all the hematologists-oncologists (100%) reported adequate adherence to ordering and evaluating complete blood count with differential, kidney function tests, and liver function tests before each chemotherapy session and for follow-up. On the other hand, 50% of the hematologists-oncologists reported inadequate adherence to counseling the patients about the alarming symptoms that require immediate medical help at the beginning of the treatment and whenever there was a need. Moreover, 20% of the hematologists-oncologists reported inadequate adherence to assessing the vital signs at each encounter, measuring, and documenting the body mass index at least once at the beginning of the treatment and whenever there was a change.

#### Monitoring and maintaining bone health

Of the hematologists-oncologists, 90% reported inadequate adherence to screening for medication problems related to bone health during treatment and whenever the patient complained about issues. In addition, 70% of the hematologists-oncologists reported inadequate adherence to ordering and evaluating calcium, vitamin D, alkaline phosphatase, electrolytes, and phosphorus levels to monitor bone health and referring patients to orthopedic surgeons for vertebroplasty, kyphoplasty, or surgical fixation when there was a need. Moreover, 60% of the hematologists-oncologists reported inadequate adherence to prescribing calcium and vitamin D supplements whenever there was a need and 50% of the hematologists-oncologists reported inadequate adherence to prescribing bisphosphonates at the beginning of the treatment, when there were no contraindications, and whenever there was a need.

#### Monitoring and managing pain

In this study, 90% of the hematologists-oncologists reported inadequate adherence to assessing pain including neuropathy at the beginning of treatment, ordering, and evaluating physical examinations and radiologic imaging to evaluate any new pain whenever the patient complained, and whenever there was a need. In addition, 60% of the hematologists-oncologists reported inadequate adherence to prescribing medications to reduce pain and anxiety before performing painful and stressful procedures and 50% of the hematologists-oncologists reported inadequate adherence to prescribing analgesics including narcotics to manage pain whenever there was a need.

#### Monitoring mobility, physical activity, mental health, and well-being

In this study, the vast majority of the hematologists-oncologists (90%) reported inadequate adherence to assessing physical activity habits and adherence of the patients to healthy behaviors. Similarly, the vast majority of the hematologists-oncologists did not screen for alcohol and tobacco use, cardiopulmonary fitness, fatigue, emotional distress, and nutritional deficits. Moreover, the vast majority of the hematologists-oncologists did not prescribe a tailored physical activity plan for the patients at the beginning of treatment and whenever there was a need. Of the hematologists-oncologists, 60% reported inadequate adherence to assessing arthritis and musculoskeletal issues or recommended the use of a cane or a walker whenever there was a need. Adherence of the hematologists-oncologists to the recommended guidelines for monitoring patients with multiple myeloma is shown in Table 3.

**Table 3:** Adherence of the hematologists-oncologists to the recommended guidelines for monitoring patients with multiple myeloma

		Adherence to the recommendations/guidelines				
		Inadequate adherence		Adequate adherence		
#	Item	n	%	n	%	References
	**Monitoring vital signs, hematologic issues, kidney, and liver functions**					
1	Assess the vital signs at each encounter, measure, and document the body mass index at least once at the beginning of the treatment and whenever there is a change	2	20	9	90	[1, 10]
2	Order and evaluate complete blood count with differential to screen for hematologic issues including anemia, neutropenia, and thrombocytopenia before each chemotherapy session and for follow-up	0	0	10	100	[10, 16, 29–32]
3	Order and evaluate kidney function tests and liver function tests before each chemotherapy session and for follow-up	0	0	10	100	[10, 16, 29–32]
4	Counsel the patients about the alarming symptoms that require immediate medical help at the beginning of the treatment and whenever there is a need	5	50	5	50	[10, 16, 29, 30, 32]
	**Monitoring and maintaining bone health**					
1	Order and evaluate calcium, vitamin D, alkaline phosphatase, electrolytes, and phosphorus levels to monitor bone health at the beginning of the treatment and for follow-up	7	70	3	30	[6, 10, 37, 38]
2	Prescribe calcium and vitamin D supplements whenever there is a need	6	60	4	40	[10, 37]
3	Prescribe bisphosphonates at the beginning of the treatment, when there are no contraindications, and whenever there is a need	5	50	5	50	[10]
4	Screen for medication problems related to bone health during treatment and whenever the patient complains about issues	9	90	1	10	[14, 39]
5	Refer patients to orthopedic surgeons for vertebroplasty, kyphoplasty, or surgical fixation when there is a need	7	70	3	30	[10, 40, 41]
	**Monitoring and managing pain**					
1	Assess pain including neuropathy at the beginning of treatment, whenever the patient complains about pain, and whenever there is a need	9	90	1	10	[2, 3]
2	Order and evaluate the physical examination and radiologic imaging (i.e., x-ray, magnetic resonance imaging, positron-emission tomography, computed tomography, bone survey, and bone density tests) to evaluate any new pain and whenever there is a need	9	90	1	10	[16, 30–32]
3	Prescribe analgesics including narcotics to manage pain whenever there is a need	5	50	5	50	[4, 5]
4	Prescribe medications to reduce pain and anxiety before performing painful and stressful procedures whenever there is a need	6	60	4	40	[46]
	**Monitoring mobility, physical activity, mental health, and well-being**					
1	Asses physical activity habits and adherence to healthy behaviors at the beginning of treatment and whenever there is a need	9	90	1	10	[39, 42]
2	Asses alcohol and tobacco use at the beginning of treatment and whenever there is a need	9	90	1	10	[39]
3	Asses cardiovascular and pulmonary fitness at the beginning of treatment and whenever there is a need	9	90	1	10	[14, 16, 29, 30, 39]
4	Screen for fatigue and its contributing factors including endocrine, electrolyte, sleep, and other dysfunctions at the beginning of treatment and whenever there is a need	9	90	1	10	[39, 43]
5	Asses arthritis and musculoskeletal issues including steroid myopathy at the beginning of treatment, during treatment, and whenever the patient complains about issues	6	60	4	40	[16, 30, 32, 45]
6	Screen for emotional distress including depression and anxiety whenever the patient complains about issues and whenever there is a need	9	90	1	10	[44, 45]
7	Screen for nutritional deficits and refer patients to a nutritionist at the beginning of treatment, whenever the patient complains about issues, and whenever there is a need	9	90	1	10	[14, 39]
8	Prescribe a tailored physical activity plan to help maintain balance, strength, and fitness at the beginning of treatment and whenever there is a need	9	90	1	10	[39, 42, 43]
9	Recommend patients to use a cane or walker to help them with mobility whenever there is a need	6	60	4	40	[10]

## Discussion

Because of the increased risk of pathological fractures, caring for the bone health of patients with multiple myeloma is crucial [4, 5]. Additionally, the different treatment modalities used to manage multiple myeloma like chemotherapy and glucocorticoids also increase the risk of bone fractures [49]. Therefore, it is important to assess and manage the bone health of patients with multiple myeloma to prevent fractures, maintain their quality of life, and improve their overall health outcomes [5, 10, 14–17]. In this study, adherence of the hematologists-oncologists to the consensus/evidence-based recommendations for assessing, managing, and monitoring bone health, pain, and mobility in patients with multiple myeloma who received care in the Palestinian healthcare system was assessed for the first time. Decision-makers and policymakers in the Palestinian healthcare system and those in other poor, developing, and resource-limited settings might benefit from the findings reported in this study to design measures and implement policies to increase adherence to the consensus-based guidelines and recommendations while caring for patients with multiple myeloma.

The findings of this study showed inadequate adherence to the recommended guidelines for diagnosing patients with multiple myeloma, particularly ordering and evaluating blood calcium levels and bone imaging to look for osteolytic lesions. It is worth noting that blood calcium levels and osteolytic lesions are important in the diagnosis of multiple myeloma as per the revised IMWG diagnostic criteria [1, 10, 16, 29–36]. It is well-established that multiple myeloma is associated with hypercalcemia [50]. Hypercalcemia is known to cause kidney disease, increase the risk of bone fractures, and decrease the effectiveness of certain chemotherapeutic agents. Despite advancements in diagnostic and therapeutic options, hypercalcemia remains an important adverse prognostic factor that should be considered while diagnosing new cases of multiple myeloma [29–36, 38, 48, 50]. Because these tests are essential for accurately diagnosing and staging multiple myeloma, failure to perform these tests could lead to missed diagnoses and/or suboptimal treatment. In a previous study, the Poitou-Charentes cancer registry was used to assess adherence to the recommended guidelines for the diagnosis, staging, treatment, and prognosis of patients with multiple myeloma [51]. Adherence to the recommended guidelines for diagnosing patients with multiple myeloma was 98%. In Palestine, caring for cancer patients has long been described as fragmented [52, 53]. Lack of coordination between hematologists-oncologists, radiologists, pathologists, and surgeons was previously reported [52]. The 5 centers where patients with multiple myeloma receive healthcare are small hematology/oncology units. These centers lack specialized pathology laboratories, imaging, and advanced diagnostic facilities. Currently, there is a need for a comprehensive care center offering all diagnostic, treatment, and care services to patients with multiple myeloma.

In this study, the hematologists-oncologists reported inadequate adherence to the recommended guidelines, notably monitoring and maintaining bone health. Because osteolytic lesions and fractures are very common among patients with multiple myeloma [7, 13], hematologists-oncologists should periodically monitor and maintain bone health. This can be done by screening for medication problems, maintaining optimal calcium and vitamin D levels, prescribing bisphosphonates, and referring patients to orthopedic surgeons whenever needed. It is noteworthy to mention that multiple myeloma can comorbid with osteoporosis in older adults [6]. Moreover, many of the medications used to treat multiple myeloma like corticosteroids can increase the risk of bone loss and fractures [10, 54]. Similarly, optimal calcium and vitamin D levels should be maintained. In the absence of contraindications, bisphosphonates, calcium, and vitamin D supplements should be prescribed to patients with multiple myeloma [10]. Moreover, the findings of this study have shown that there was inadequate adherence to assessing and managing pain. Pain is one of the most common and distressing symptoms experienced by patients with multiple myeloma [55]. Multiple myeloma can cause bone pain, neuropathic pain, and pain associated with fractures, and pain can also be a side effect of treatment [55, 56]. Uncontrolled pain can lead to decreased quality of life, decreased mobility, and increased risk of depression and anxiety [56]. Therefore, it is recommended that healthcare providers assess and identify the type, severity, and location of pain, and develop an individualized pain management plan that can improve patient comfort and quality of life [1]. Additionally, regular pain assessment can also help to detect new or worsening pain early, which may be a sign of disease progression or treatment side effects, allowing for timely intervention.

In this study, a lack of adherence to the recommended guidelines for the management of patients with multiple myeloma was also reported in several supportive areas, notably screening for fatigue, assessing cardiovascular and pulmonary fitness, assessing and supporting mobility, prescribing physical activity plans, screening for nutritional deficits, and maintaining the well-being of the patients. These findings were not surprising as psychosocial support, rehabilitation, palliative care, and nutritional services offered to cancer patients in the Palestinian healthcare system are substandard [52]. Inadequate addressing of these issues can result in poor health outcomes, increased risk of complications, and deteriorate the quality of life of the patients [1, 5, 10, 14, 16].

### Strengths and limitations

The findings of this study should be interpreted after considering the following strengths and limitations. First, this was the first study to assess the extent of adherence to the recommended guidelines that should be used in the assessment and management of bone health, pain, and mobility in patients with multiple myeloma in the Palestinian healthcare system. The findings of this study might reflect those in the other healthcare systems of poor, underdeveloped, and resource-limited countries. Second, the study was inclusive of all healthcare centers where patients with multiple myeloma received healthcare in Palestine. Therefore, the findings of this study are reflective of those seen in the different centers. Third, a mixed method was used in this study. Mixed methods that combine qualitative and quantitative approaches are powerful in portraying the practices followed in the healthcare system.

On the other hand, the study had some limitations. First, the sample size used in this study was very small. It is noteworthy to mention that only 5 centers in the West Bank care for patients with multiple myeloma. However, instead of asking the head of the hematology/oncology department and a main hematologist-oncologist from each center to respond to the questionnaire, we could have asked all hematologists-oncologists to participate in the study. This might have reduced the potential selection bias in this study. Purposive sampling has long been criticized as biased. Second, the answers of the hematologists-oncologists were self-reported. Therefore, desirability and recall biases could not be excluded. Second, patients were not interviewed in this study. The inclusion of patients should have added more richness to the findings reported in this study. Third, the study was conducted among hematologists-oncologists, the inclusion of other healthcare providers might have also provided richness to the findings reported in this study. Fourth, the extent of awareness of the hematologists-oncologists of the existing guidelines should have been assessed in this study. In this study, the interview guide was based on the IMWG guidelines. Furthermore, more interview questions should have been included to explore the reasons for lack of adherence to the international guidelines. This should have provided more insights into the effects of the limited number of service providers, lack of coordination, and equipment on adherence to the international guidelines. Finally, the findings of this study cannot be generalized to the entire population of hematologists-oncologists providing care services to patients with multiple myeloma in the Palestinian healthcare system.

## Conclusion

In conclusion, the findings of this study suggested inadequate adherence to the recommended guidelines for the assessment and management of bone health, pain, and mobility in patients with multiple myeloma who receive care in the Palestinian healthcare system. Overall, the study provided insights into the current practices used by hematologists-oncologists in the Palestinian healthcare system for assessing and managing bone health, pain, and mobility in patients with multiple myeloma and highlighted areas for improvement to ensure that patients receive optimal care. The findings suggest a need for further education and training on the latest guidelines and recommendations. Decision-makers and policymakers might need to design measures and implement policies to improve adherence to the recommended guidelines. Addressing these gaps in adherence to recommended guidelines may improve the care and outcomes of multiple myeloma patients.

## Data Availability

All data relevant to this study were included in the results section of this manuscript.
